# Self-assembled large scale metal alloy grid patterns as flexible transparent conductive layers

**DOI:** 10.1038/srep13710

**Published:** 2015-09-03

**Authors:** Melinda Mohl, Aron Dombovari, Robert Vajtai, Pulickel M. Ajayan, Krisztian Kordas

**Affiliations:** 1 Microelectronics and Materials Physics Laboratories, Department of Electrical Engineering, University of Oulu, P.O. Box 4500, Oulu FIN-90014, Finland; 2 Department of Material Science and Nano Engineering, Rice University, Houston, Texas 77005, United States

## Abstract

The development of scalable synthesis techniques for optically transparent, electrically conductive coatings is in great demand due to the constantly increasing market price and limited resources of indium for indium tin oxide (ITO) materials currently applied in most of the optoelectronic devices. This work pioneers the scalable synthesis of transparent conductive films (TCFs) by exploiting the coffee-ring effect deposition coupled with reactive inkjet printing and subsequent chemical copper plating. Here we report two different promising alternatives to replace ITO, palladium-copper (PdCu) grid patterns and silver-copper (AgCu) fish scale like structures printed on flexible poly(ethylene terephthalate) (PET) substrates, achieving sheet resistance values as low as 8.1 and 4.9 Ω/sq, with corresponding optical transmittance of 79% and 65% at 500 nm, respectively. Both films show excellent adhesion and also preserve their structural integrity and good contact with the substrate for severe bending showing less than 4% decrease of conductivity even after 10^5^ cycles. Transparent conductive films for capacitive touch screens and pixels of microscopic resistive electrodes are demonstrated.

Due to their outstanding light transmission, and excellent electrical conductivity transparent conductive electrodes, also known as transparent conductive films (TCFs), are widely applied in smart phones, touch screens, light–emitting diodes and thin-film solar cells^1^,^2^,^3^,^4^. The most prevalent materials applied as TCFs in both polymer and inorganic solar cells are aluminum-doped zinc oxide (AZO) and indium tin oxide (ITO), having high optical transparency (*T*), reaching of about >90%, and a sheet resistance (R_s_) as low as ~10 Ω/sq^5^,^6^, and synthesized by chemical sol-gel methods as well as physical deposition/growth techniques such as e-beam evaporation and sputtering^1^,^7^. However, a progressively developing disadvantage of applying ITO is lying in the scarceness of indium resources which is soon expected to eventuate in increased market price and shortcoming of ITO supplies^8^,^9^,^10^. Moreover, the brittleness^8^,^9^,^10^ of ITO films severely limits its use in flexible applications, gradually emerging nowadays; therefore alternatives to replace ITO in future transparent and conductive applications are timely and have been subjected to intensive research for a decade^11^,^12^,^13^. Although a significant number of candidate materials have been arising, such as carbon nanotubes (CNTs)^14^, metal nanowires^15^,^16^,^17^,^18^,^19^, graphene^20^, polymers^11^, alternative metal oxides^21^,^22^ and their hybrids/composites^23^,^24^,^25^, currently none of those can meet all the standards of prospective devices^6^. It is generally agreed that the material substituting ITO should have R_s_ < 100 Ω/sq, though the specifications highly depend on the particular application^6^,^26^,^27^,^28^. For example, touch screens require *T* higher than 95% while allow R_s_ of 400–600 Ω/sq^26^; meanwhile solar cells and flat panel displays demand R_s_ values lower than 20 Ω/sq^27^,^29^,^30^. In addition, future devices envisaged to be prepared on flexible plastic surfaces, thus, transparent electrodes with elasticity, printability coupled with cost-effective low temperature fabrication technology, could open up opportunities in several areas of modern electronics. Researchers have thus far focused on manipulating materials/coatings to have high optical transparency and metallic conductivity, an inherently contradictory attribution in one material. Recently, various types of networks of metal nanostructures^31^,^32^ and carbon nanotubes^12^—either random or oriented – have been widely considered to be an ideal solution for resilient TCFs. One of the simplest way of constructing highly ordered structures is capitalizing the coffee-stain effect^33^,^34^,^35^, which is a rapid, non-lithographic, easy-to-scale and yet cheap process^36^. Although much effort has been devoted to study this everyday phenomenon, most of the previous studies have been limited to understand the physical effects responsible for the ring formation^33^,^35^; nevertheless, some attempts have been made to assemble metal nanoparticles into a conductive system by exploiting drying droplets of metal nanoparticle dispersions in order to prepare transparent conductive coatings^32^,^37^.

Here we show a novel method to obtain periodic ring patterns of AgCu by using reactive inkjet printing and subsequent chemical copper plating. We further demonstrate printed grid meshes from palladium reactive ink plated with copper to create high performance transparent and conductive electrodes. These printed films possess outstanding electrical properties having sheet resistance values well below 10 Ω/sq while allowing optical transmittance close to 80% at 500 nm.

Inkjet deposited solid circle shaped drops (~80 μm in diameter) and coffee-rings (with rim width of 6–10 μm) can be deposited from a palladium and silver precursor solutions, respectively, to seed the surface for subsequent chemical plating with copper. First, the poly(ethylene terephthalate) (PET) substrate is treated by using argon plasma (Fig. 1) in order to enable well controlled formation of coffee-rings and solid circles on the otherwise hydrophobic surface. In the next step, the water based metal precursor inks are inkjet deposited onto the polymer film. Subsequently, the palladium precursor pattern is heated at 200 °C in air for 10 minutes in order to decompose Pd(OAc)_2_ while in the case of silver patterns such treatment is not necessary since diamminesilver(I) ions are reduced by glucose in the course of drying on the heated platen. In the final step, the catalyst seeded substrates are placed into a copper plating bath for 4 minutes to develop thin copper films on the patterned surface.

PdCu grid patterns have been prepared in six different sizes having inner unprinted squared areas with sides of approximately 60, 120, 180, 300, 600 and 900 μm, whereas the diameter of the disks, interconnecting as conductive lines, is about 60 μm. Typically, the width of the printed lines depends on the pretreatment, therefore in reality the size of the unprinted areas are slightly smaller compared to the printing pattern (Fig. SI-5). The area density of conductive lines can be easily adjusted by altering the printing pattern. AgCu coffee-stain diameter varies from 72 to 81 μm while width of the rims was found to be about 6–10 μm, as assessed by electron microscopy analysis.

When printing patterns, it is important to apply a proper drop deposition sequence to avoid merger of droplets. This has been achieved by optimizing the drop spacing so that the independent deposited droplets abut only at the rim. Addition of 2,3-butanediol to the reactive inks enables adjusting the viscosity, an important printing parameter, and it also contributes to the formation of coffee-rings in the case of silver ink. Viscosity and density values for both inks are summarized in Table SI-1.

PET films, as most of the polymers, are known to be rather hydrophobic that can have significant influence on the adhesion of surface coatings and subsequent patterning processes. In the absence of substrate treatment small liquid beads evolve via coalescence of printed droplets exhibiting weak adhesion after drying. Even though chemical etching is one prevalent way to modify the surface roughness and to improve wetting, attempts printing on hydrolyzed^38^ PET did not lead to the expected results. Therefore in advance of printing, argon plasma treatment was performed in order to reduce the inherent hydrophobic nature of the substrate and allow the formation of homogeneous patterns by having control on the droplet size and, consequently, on the formation of the final pattern. Furthermore, as the modified surface of plasma-treated polymers tends to recover in virtue of the re-orientation of induced polar functional groups^39^ the optimum aging time of surface modification before printing and copper plating have been studied (Fig. SI-5). As might be anticipated the more time elapsed subsequent to plasma treatment the more contracted the seeding droplets were – as the surface progressively changed from hydrophilic to hydrophobic. The diameter of the solid discs of PdCu was observed to be ~30% smaller when printed on the 7th day compared to processes done on the 1st day after treatment (Table SI-2). We note that our observations are in good agreement with some earlier results^39^ that the contact angle of water on argon plasma treated PET is rapidly increasing in the first 10 days of storage, similar to a saturation curve, whereas in the next 15 days it reaches a plateau slightly under the value of the untreated substrate. In the case of silver-copper patterns the changes were far less conspicuous (Table SI-3), however, a slight decrease in the drop diameter, from 81 to 72 μm, was observed when printing was performed a couple of days after the plasma treatment. Moreover, in the lack of surface treatment coffee-rings were not formed instead smaller solid droplets evolved. As expected, the time passed amid printing and plating was found to be indifferent for the size of the copper features since the deposition of copper is triggered by the printed seeds.

Figure SI-1 and SI-2 show top views of the PdCu and AgCu patterns, respectively, in different magnification. Obviously, the PdCu structure consists of mostly uniformly distributed grains with a size of less than 200 nm. Interestingly, at the junction of separate droplets the grain size of the metal structures is somewhat smaller (upper part on Fig. SI-1 d-f). Needless to say that this technical issue can simply be solved by printing two layers of separate droplets, however no difference in electrical performance was observed between these films. For AgCu structures (Fig. SI-2) grains of copper form a more homogeneous coating that we believe is caused by the simultaneous growth of copper initiated on a higher concentration to area of silver seeds compared to palladium that can be explained by (i) the more than 2 times greater initial concentration of metal in the reactive silver ink, (ii) the faster generation of seeds, (iii) the higher printing plate temperature, and (iv) the congregation of seeds at the rims. The majority of the particles (Ag and Pd) have a size (not shown) smaller than 50 nm, although some of them are aggregated, thus the size can be up to several hundreds of nanometers. On the EDX maps (Fig. SI-3) both seeds of palladium and silver are slightly visible, while on the XRD patterns (Fig. SI-4) the only distinguishable reflections are attributed to copper and PET. Thickness of the printed films after copper plating, determined by atomic force microscopy (AFM), has been found to be between 200–250 nm (Fig. 1c,d).

In the case of the PdCu grid patterns, the sheet resistance values were found to vary between 0.8 and 8.1 Ω/sq (4-point probe data) with corresponding optical transmittances between 31.0 and 78.8% at 500 nm (Fig. SI-6), depending on the size of the holes in the pattern. As for the AgCu fish scale-like structures, the ring-shape formation from the silver ink were found to be very sensitive to the process parameters. After electroless copper plating, self-similar Cu patterns with 2.0–34.4% transmittance and corresponding sheet resistance between 0.6–24.4 Ω/sq were obtained. However, one problem encountered with these films, revealed by using electron microscopy and energy-dispersive X-ray spectroscopy (Fig. SI-7) that a small amount of seeding material may remain inside the rings and can initiate undesirable copper deposition at the plating process. This phenomenon could explain the large deviation in the performance between the metal electrodes. We have addressed this issue by inserting one further step (Fig. 1) after copper plating. Undesirable deposits of copper can be easily removed selectively from the inner area of the rings by using sodium persulfate (Na_2_S_2_O_8_) solution, generally applied for pickling copper, which etching resulted in a substantial increase in the transmittance, at the same time, the ring structure remained unaffected (Fig. 2). By increasing the etching time from 20 to 40 and 60 s the optical transmittance increased (Fig. SI-8) accordingly to 42, 50, and 65%, respectively. Unsurprisingly, partial removal of the copper coating is accompanied with some loss in the conductivity, which in the latter cases resulted in R_s_ of 3.2, 2.8, and 4.9 Ω/sq, respectively. Please note that the initial Rs and T values of the samples were different.

In order to assess the specific conductivity of the PdCu electrodes, straight lines were deposited on the polymer. The thickness of the lines was determined by AFM measurements (Fig. 3) and current-voltage (I-V) curves (Fig. 3) were used to calculate the resistivity. The exact geometry, width and length, was analyzed by using optical images. The specific conductivity was found to be 2.0 ± 0.7 × 10^7^ S/m which is quite high considering the specific conductivity of bulk copper (5.84 × 10^7^ S/m at 298 K).

Both transparent electrodes demonstrated superb structural integrity during mechanical impacts of different types (Fig. 3a,b). Repeated Scotch tape tests performed on the metal patterns could not remove the conductive coatings from the surface, and only caused a slight increase in the sheet resistance values (PdCu grids) or had no effect at all (AgCu patterns) proving their excellent adhesion to the PET substrate (Table SI-4).

To quantitatively evaluate the impact of bending on the electrical conductivity, the sheet resistances of PdCu and AgCu coatings were measured after successive bending cycles (Fig. SI-13 and Video SI-1), as was done previously for films composed of carbon nanotubes^40^, Ag^5^ and NiCu^41^ nanowires on flexible polymer substrates. Figure 3b shows that both types of films could be significantly bent without damage and severe increase in the sheet resistance. After 10^5^ bending cycles, the sheet resistance of the metal films increases with only less than 4% for tensile and 5% in case of compressive bending for both PdCu and AgCu, demonstrating a remarkably robust flexibility clearly outperforming other known transparent conductive coatings.

The sheet resistance of different type of TCFs in the function of optical transmittance, based on the references in Table SI-5–8, is shown on Fig.SI-14. Substantial effort has been placed on developing TCFs of carbon nanotube networks^42^ and graphene^43^, yet most of these films suffer from high sheet resistance originating from the charge transport barrier formed at the surface of the particles in contact. Nevertheless, unique properties of graphene^44^ make it suitable to replace ITO in case the production of large area single piece of monolayers becomes feasible at an affordable price. Although the manufacturing processes of conductive polymer-based electronic appliances include generally rather simple and inexpensive methods, so far owing to their quite high Rs values^45^,^46^ polymer films are still not able to fulfill the current requirements of TCFs. Currently, the best performance similar to ITO has been achieved by using metal structures^47^,^48^,^49^ or combination of the separate alternatives/ hybrid films^50^,^51^.

Although the manufacturing processes of large–area electronic applications are very often rely on polymer coatings since their preparation is rather simple and can be performed at room temperature, yet owing to their well-defined and wide range of electrical properties inorganic building materials in electronic appliances are still more popular^52^. Nevertheless, the currently applied techniques for inorganic thin films are costly and arduous thus, simple and low-priced approaches allowing direct micropatterning and low temperature processing are in great demand. Additive methods, as inkjet printing, enable simultaneous/subsequent deposition of a large variety of materials, easy prototyping and scalable production. As illustrated by Johannes Gutenberg’s portrait printed on a PET film (Fig. 4), patterns of practically any kind at rather low temperature may be produced by using the PdCu method.

To further elaborate on the practical aspects of the methods reported here, we demonstrate functional micropatterns of the PdCu thin films such as capacitive touch screen (Fig. 5a–d) and microscopic interdigitated planar electrodes (Fig.5e–h). Using grid structures we show reliable switching operation by turning on/off a light-emitting diode simply touching the polymer bottom of the substrate using a finger (Video SI-2). On the other hand, interdigitated microscopic electrodes deposited on the PET substrate allow resistive switch operation (Video SI-3). When touching the electrodes under a.c. bias (in our case 1 V at 100 kHz), the originally high impedance capacitive planar electrode structure (Z ~30 MΩ and φ ~ −90°) is turning to a resistive component with a significantly decreased impedance of ~100 kΩ.

In this article, we demonstrated a facile and easy to scale-up method to prepare TCFs by simply combining reactive inkjet printing with the well-known coffee-ring effect to form microscopic patterns of copper on polymer surfaces. Studies focusing on the preparation of flexible printed circuitry by inkjet printing is rapidly emerging^53^ although, to our knowledge the combination of self-aligned patterning^37^, reactive ink printing^53^,^54^,^55^, and subsequent chemical plating of copper^53^, has not been applied before to achieve TCFs with superior quality. Our method exploits the advantages of the above listed techniques resulting in highly conductive and transparent coatings with excellent reliability. The demonstrated technology paves the route towards cost-effective practical replacement of ITO coatings and may be developed even further to comply with roll-to-roll processes allowing truly large-scale production of TCF components with high lateral resolution (500 dpi) for a number of different applications including capacitive and resistive touch screens.

## Methods

### Preparation of transparent metal electrodes

Plasma etching (Oxford Instruments Plasmalab 80 Plus) of PET (Melinex ST506/505) substrates was applied for 5 minutes with the conditions of 20 W and 60 W of plasma power, argon gas flow rate of 20 sccm at 20 mTorr of pressure. (Droplet diameter size ranged from ~80 μm to ~100 μm for plasma treatment of 20 and 60 W, respectively.)

Printing was performed using a piezoelectric Dimatix, DMP-2800 inkjet printer using a waveform of A3.6 P3.7 R3.4 D0.8, and A3. 6 P3.7 R20.6 D0.8 (A = attack (μs), P = pulse (μs), R = release (μs), D = Delay (μs)) for silver and palladium ink, respectively. Further printing parameters of Pd and Ag patterns are summarized in Table SI-9, and Table SI-10, respectively. For *palladium reactive ink* 22.45 mg of Pd(OAc)_2_, and 6.0 mg of NaOH were dissolved in a mixture of 0.6 mL NH_3_ (25%), and 1.4 mL distilled water. The solution was kept in a dark place for overnight before the addition of 2.5 mL of HCOH (35–40%), and 0.5 mL of 2,3-butanediol. *Reactive silver ink* was prepared by dissolving 27.2 mg of AgNO_3_ in 0.125 mL distilled water, subsequently 0.375 mL NH_3_ (25%), and 1.5 mL of glucose solution (0.1 M) were added, followed by 1.0 mL of 2.3-butanediol.

PET substrates with printed Pd electrodes were placed in a box furnace at the following temperatures 100; 125; 150; 175; 200; 220 °C for 1; 8; 10; 15 minutes. For an ideal copper coating 10 minutes at 200 °C were determined as optimal treatment. After the chemical copper plating step, the films were immersed into DI water to remove the excess of plating solution from the surface.

*Copper plating solution* was freshly made before the plating process by dissolving 0.5 g CuSO_4_, 3.3 g KNaC_4_ H_4_O_6_ × 4H_2_O, and 0.5 g NaOH in 50 mL DI water. Finally 2.6 mL of HCOH (35–40%) was added to the solution. The interval of copper plating was tested in the range of 2–20 minutes. To reach an electrically conductive thickness of copper without sacrificing optical transmittance the optimum duration of electroless plating was found to be 4 minutes.

In order to increase the optical transmittance of AgCu patterns samples were immersed into various liquids after printing. For washing the following liquids were applied: Soapy water (Fairy original dish soap), hot water (~50 °C), acetone, abs. ethanol, and 2-propanol (Fig SI-9 and 10). Some samples were post-treated after copper plating with nitric acid solution (SI-11 and 12) and sodium persulfate solution having a concentration of 10 mL/L, 25 mL/L, 50 mL/L, and 1 g/L, 5 g/L, and 10 g/L, respectively. The samples were immersed into the solution for 20 to 300 seconds.

### Characterization

The structure of the printed patterns was visualized by an Olympus BX51 optical microscope. For electron microscopy measurements (FESEM, Zeiss ULTRA plus at 5 kV) a small piece of PET substrate with transparent electrodes was mounted on a SEM sample holder. Elemental composition data were collected from the same samples using energy-dispersive X-ray spectroscopy at 15 kV (Inca, Oxford Instruments).

Atomic force microscopy (Veeco Dimension 3100 AFM) was performed in tapping and contact mode to perform images, and to determine the thickness of the printed lines, respectively.

To assess the square resistance of the films, platinum contact pads were evaporated at the corners of square shaped printouts (3 × 3 mm^2^) using an Agar High Resolution Sputter Coater 208HR. The resistance was measured by four-probe configuration (Van der Pauw method) using a Wentworth labs probe station sourcing 1 mA current (Keithley 6221 sourcemeter) and measuring voltage (Keithley 2182A nanovoltmeter). The conductivity of the deposited Cu was determined by analyzing the geometry (using atomic force and optical microscopy) and 4-point *I-V* measurements (current source).

Optical transmission spectra were obtained using a UV-Vis-NIR spectrometer (Cary 500), with a bare PET used as reference, in the spectral range from 350 nm to 800 nm. Optical transmission of 100% refers to the transmission of untreated blank PET substrate.

X-ray diffraction (XRD) patterns were obtained from samples mounted on zero diffraction plate using a Siemens D5000 XRD instrument operating with Cu Kα radiation.

### Viscosity and density measurements

Viscosity of the inks was determined from flow rate experiments through a laboratory-made glass capillary viscometer using the Hagen–Poiseuille equation. Density was determined by using a pycnometer.

### Flexibility tests

Samples of ~5 × 7 cm^2^ size were mounted on a glass tube (radius of 15 mm) and bent using an automated translation/rolling instrument (10–10^5^ cycles, each lasting for 4.16 s).

### Scotch tape test

Commercially available double-sided Scotch Magic^TM^ tape was pressed against the films and then detached. Repetitions from 1 to 100 were applied (after every 10 cycle, the Scotch tape was replaced with a new one).

### Capacitive and resistive touch screen testing

The grid type capacitive transparent thin films (T ~70% and Rs ~3.3 Ω/sq) were interfaced with a toggle-mode touch sensor IC (Atmel AT42QT1012) that switched a light-emitting diode (EL333-2USOD) upon touching the backside of the grid electrode. The change of impedance of the resistive pixel type electrode structures was analyzed at 100 kHz and 1V bias using an LCR meter (Hewlett Packard, 4284A).

## Additional Information

**How to cite this article**: Mohl, M. *et al*. Self-assembled large scale metal alloy grid patterns as flexible transparent conductive layers. *Sci. Rep*. **5**, 13710; doi: 10.1038/srep13710 (2015).

## Supplementary Material

Supplementary Information

Supplementary Movie S1

Supplementary Movie S2

Supplementary Movie S3

## Figures and Tables

**Figure 1:**
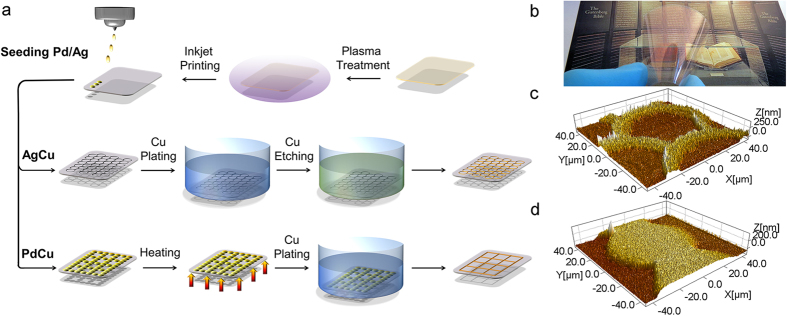
Schematic image about the preparation of AgCu and PdCu flexible conductive patterns (a) and photo of a flexible transparent PdCu pattern (b). AFM images of AgCu (**c**) and PdCu pattern (**d**).

**Figure 2:**
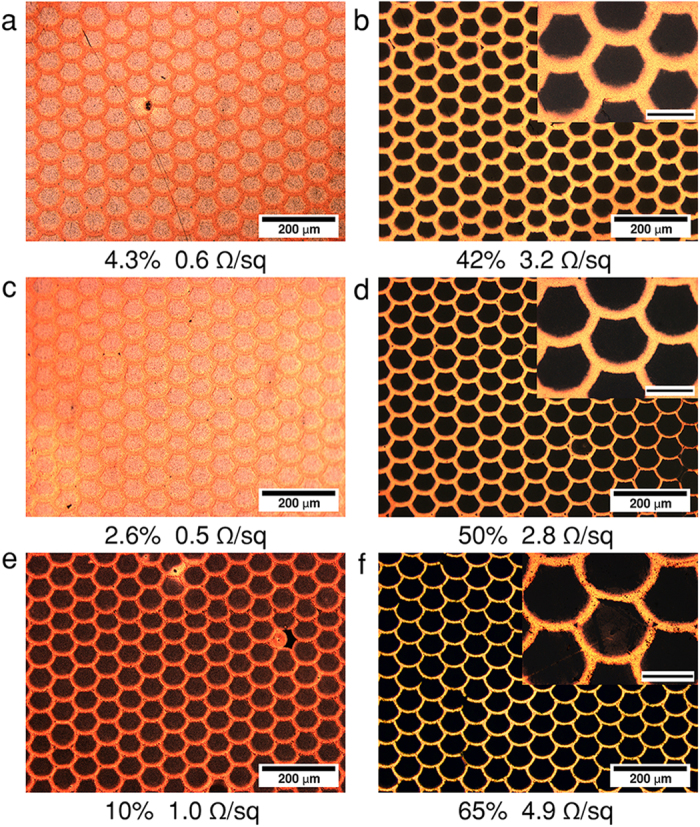
Control sample (a,c,e) and samples etched by using Na_2_S_2_O_8_ (5 g/L) for 20 s (b), 40 s (d), and 60 s (f). The scale bars in the insets are 50 μm. (Corresponding UV-VIS spectra are shown on Fig. SI-8.).

**Figure 3:**
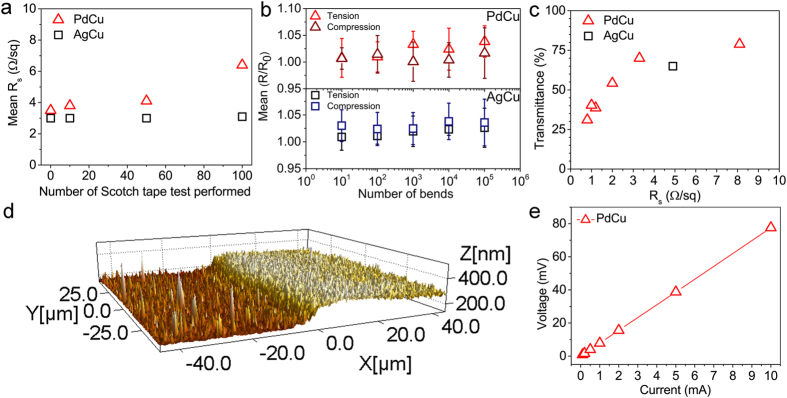
Sheet resistance in the function of number of Scotch tape test performed on the transparent metal electrodes (a), mean R_s_/R_0_ values after bending test for AgCu and PdCu patterns (b), optical transmittance as a the function of sheet resistance for PdCu grid patterns having various grid size and for AgCu pattern (c). AFM image (**d**) and I-V curve (**e**) for a PdCu straight line.

**Figure 4:**
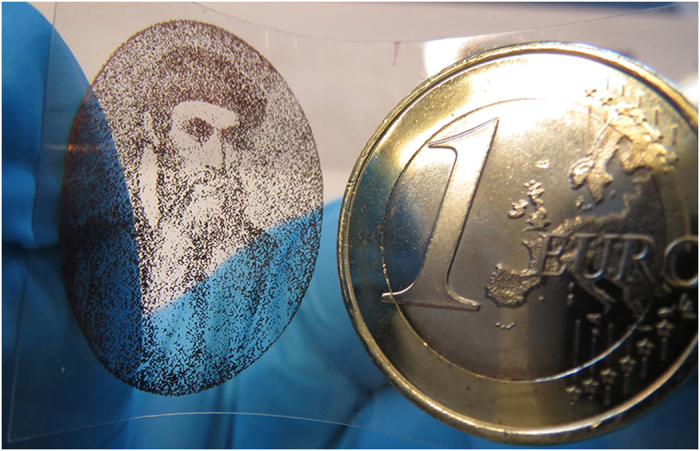
Johannes Gutenberg’s portrait inkjet printed on a PET film by using the PdCu method.

**Figure 5:**
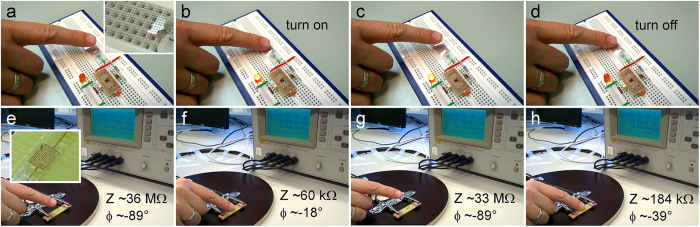
Digital camera images of a transparent conductive capacitive screen (a–d) and a microscopic resistive touch pixel (e–h) under operation. (Structure schematics of capacitive touch screen and microscopic resistive touch pixel are shown on Fig. SI-15).
