# Supplementary material for: Self-assembled large scale metal alloy grid patterns as flexible transparent conductive layers
Source: Sci Rep. 2015 Sep 3;5:13710. doi: 10.1038/srep13710 (PMC4558545; doi:10.1038/srep13710)
Supplement: Supplementary Information [file srep13710-s1.pdf]

Supplementary Information

Self-assembled large scale metal alloy grid patterns as flexible transparent conductive layers

Melinda Mohl, Aron Dombovari, Robert Vajtai, Pulickel M. Ajayan, Krisztian Kordas

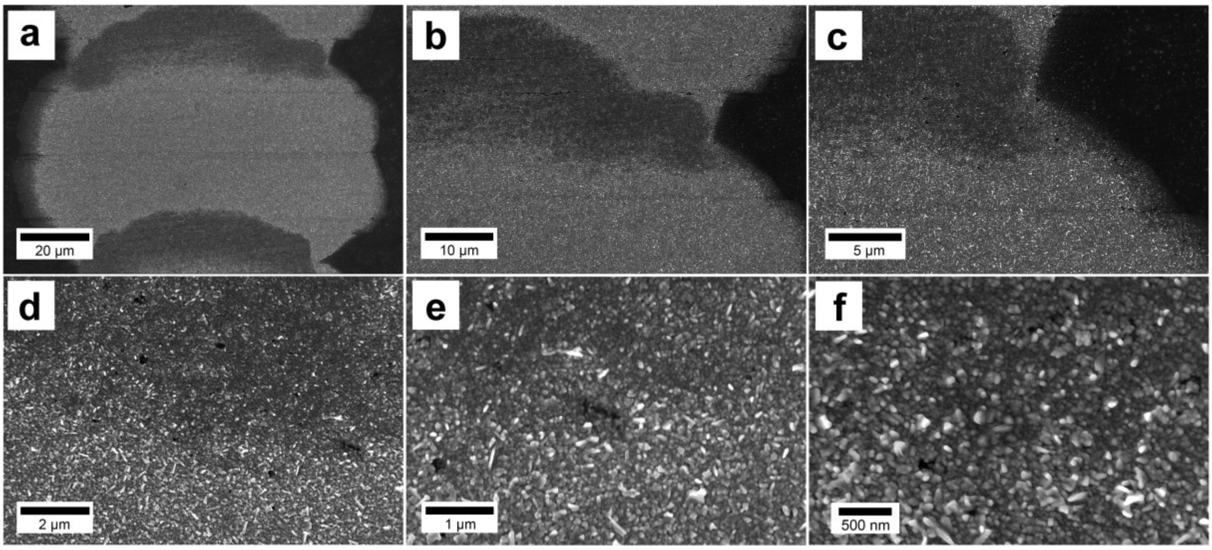

Figure SI-1 SEM images of PdCu grid patterns on PET in different magnification.

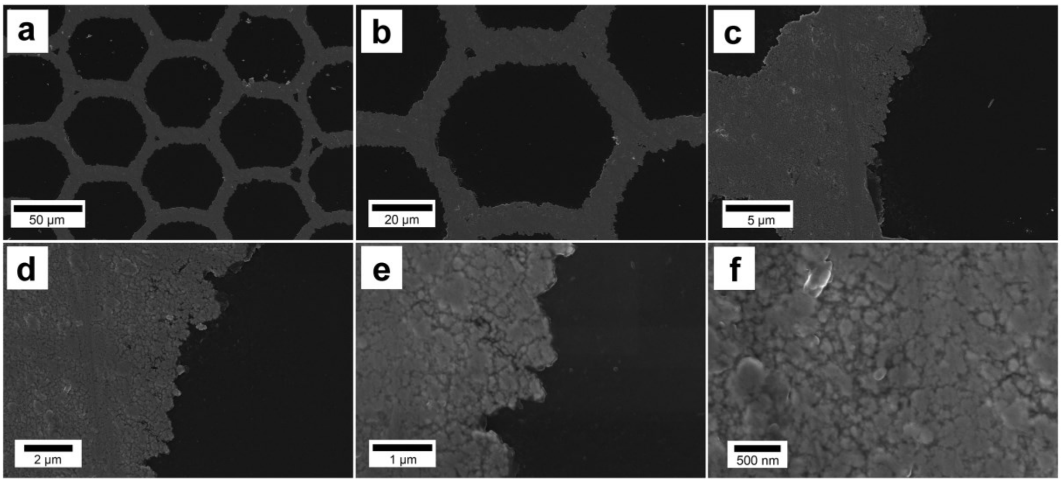

Figure SI-2 SEM images of AgCu grid patterns on PET in different magnification.

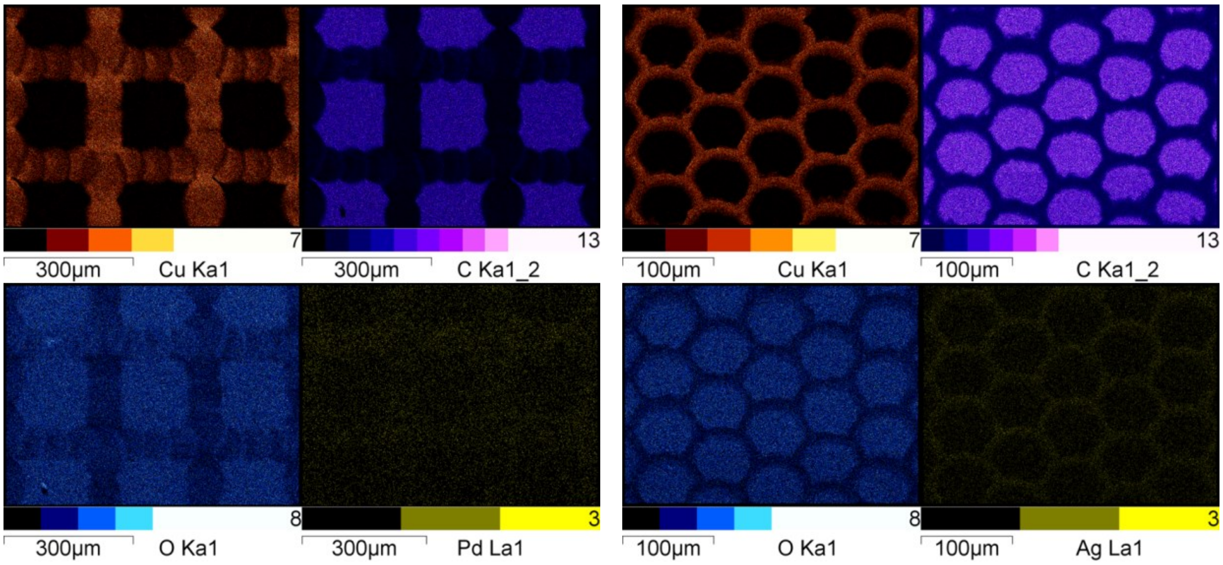

Figure SI-3 EDX maps of PdCu and AgCu patterns on PET.

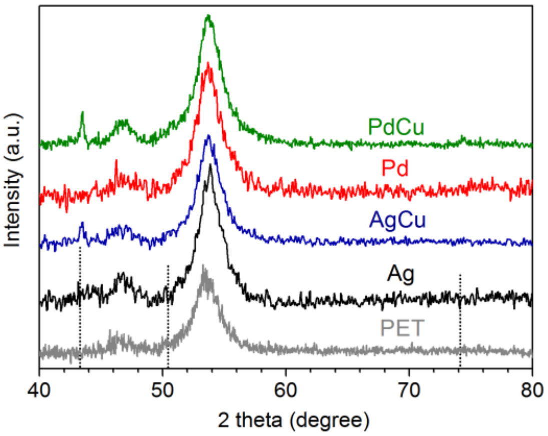

Figure SI-4 XRD patterns of PET, printed seeds (Ag and Pd), and copper plated patterns (AgCu and PdCu) on PET. Dotted black lines indicate the most intense reflections of copper (ICDD card No. 04-0836) at 43.295, 50.431, and 74.127 2 theta degree.

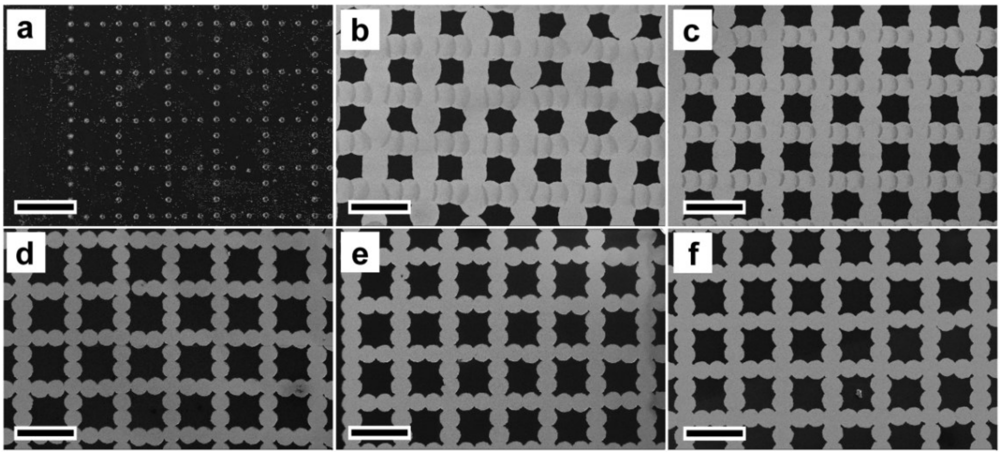

Figure SI-5 SEM images of PdCu grid patterns on PET (a) without plasma treatment, and after plasma treatment treatment: (b) 0 day, (c) 1 day, (d) 7 days. Printing 7 days after plasma treatment with copper plating 1 day after (e), and 2 days after printing (f). Scale bar 200 μm.

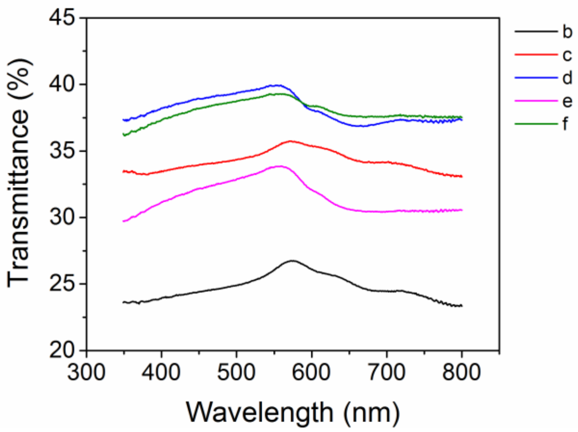

Figure SI-6 UV-VIS spectra of PdCu grid patterns on PET (a) without plasma treatment, and after plasma treatment treatment: (b) 0 day, (c) 1 day, (d) 7 days. Printing 7 days after plasma treatment with copper plating 1 day after (e), and 2 days after printing (f). Scale bar 200 µm.

Table SI-1 Viscosity and density data for silver and palladium ink.

	**Viscosity (mPa×s)**	**Density (g/cm^3)**
Silver ink	3.08	1.02
Pd ink	3.4	1.05

Table SI-2 The mean diameter size of the droplets for PdCu pattern printed and copper plated on different days after plasma treatment.

		**Cu Plating (days)**				
		**No plasma**	**0**	**1**	**2**	**6**
		**Mean diameter (µm) ± st. dev.**				
**Printing (days)**	**No plasma**	18.7 ± 1.0				
	**0**		92.0 ± 5.0	85.3 ± 1.5	86.1 ± 1.4	
	**1**		82.1 ± 2.3		82.1 ± 1.2	82.6 ± 1.4
	**2**		89.3 ± 2.4			
	**7**		62.2 ± 2.8	68.0 ± 4.4	68.3 ± 4.5	

Table SI-3 The mean diameter size of the droplets for AgCu pattern printed and copper plated on different days after plasma treatment.

		**Cu Plating (days)**			
		**No plasma**	**0**	**1**	**2**
		**Mean diameter (µm) ± st. dev.**			
**Printing (days)**	**No plasma**	56.3 ± 5.6<br>no ring formation			
	**0**				
	**1**		80.7 ± 2.9		
	**2**		72.2 ± 2.3		
	**7**		72.2 ± 3.9	74.1 ± 2.5	74.5 ± 2.3

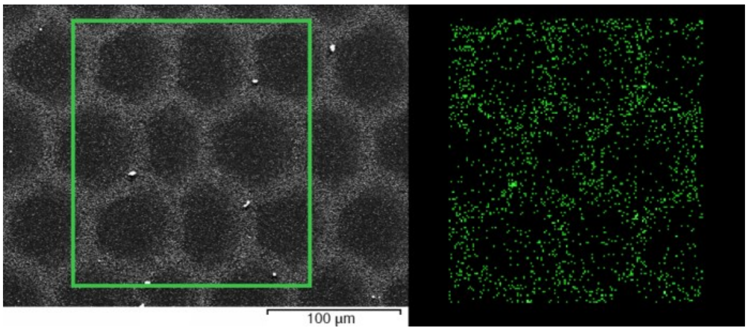

Figure SI-7. EDX AgCu pattern, showing the presence of copper also inside the areas surrounded by the ring shape patterns.

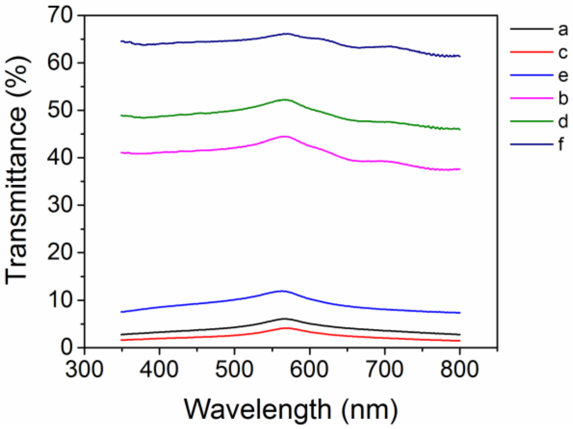

Figure SI-8. Optical transmittance of control samples (a c, e) and samples etched by using $Na_2S_2O_8$ (5 g/L) for 20 s (b), 40 s (d), and 60 s (f).

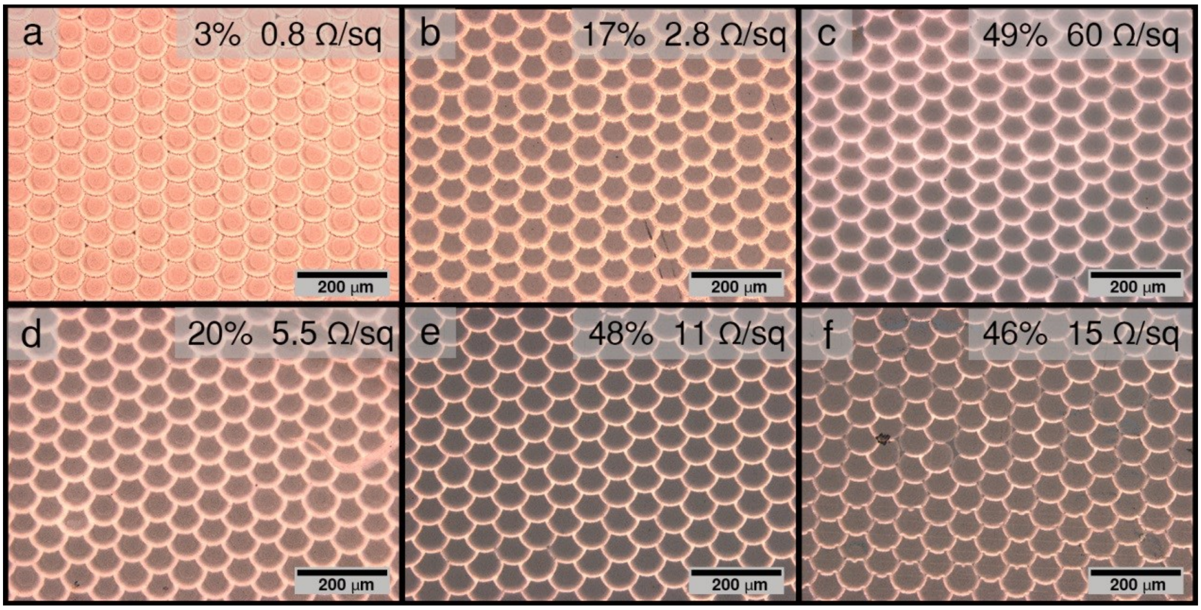

Figure SI-9 PET substrates with the inkjet printed silver seeds were immersed into different liquid phase chemicals before copper plating in order to remove residual material from the inner parts of the coffee-rings. Most of the general solvents ¬– water, aceton, isopropanol, etc. – seemed to improve the performance of the AgCu electrodes, however, none exceeded 50% of optical transmittance. Control sample (a) and samples washed after printing using hot water (b), soapy water (c), ethanol (d), 2-propanol (e), and acetone (f).

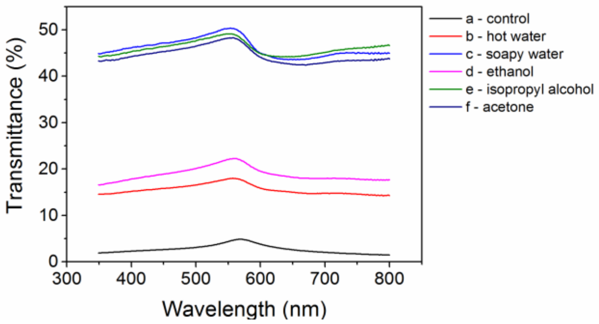

Figure SI-10 UV-VIS spectra of control sample (a) and samples washed after printing using hot water (b), soapy water (c), ethanol (d), 2-propanol (e), and acetone (f).

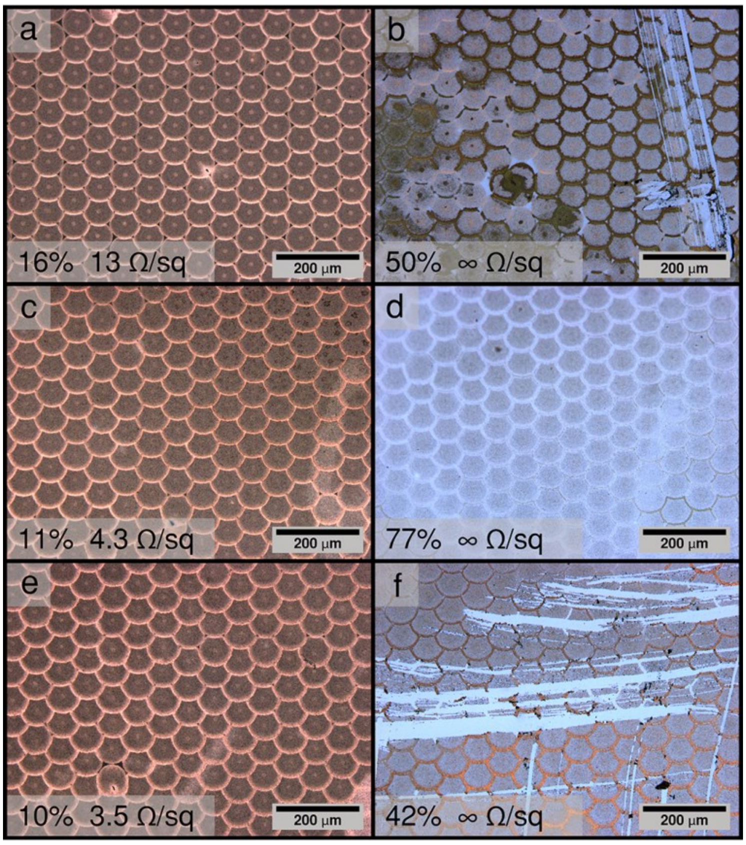

Figure SI-11 Nitric acid treatment removed the undesirable copper coating in a homogeneous manner leading to unconducive structures. Control sample (a, c, e) and samples etched by using HNO_3 (25 mL/L) for 20 s (b), 40 s (d), and 60 s (f).

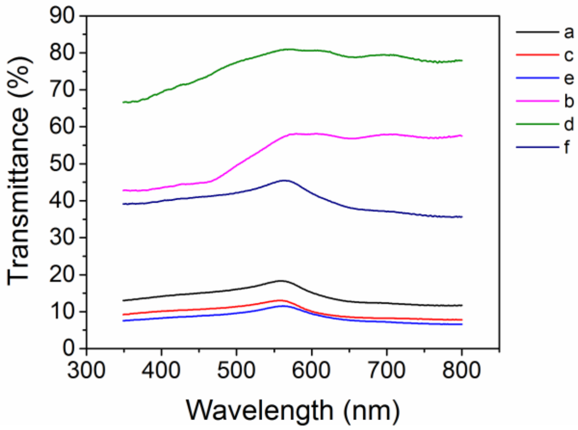

Figure SI-12 UV-VIS spectra of control samples (a, c, e) and samples etched by using HNO_3 (25 mL/L) for 20 s (b), 40 s (d), and 60 s (f).

Table SI-4 Sheet resistance values before and after scotch tape test, demonstrating the excellent adhesion of the metal electrodes to the PET substrate.

Number of scotch tape test	PdCu pattern Mean Rs (Ω/sq) ± st. dev.	AgCu pattern Mean Rs (Ω/sq) ± st. dev.
0	3.5 ± 0.03	3.0 ± 0.06
10	3.8 ± 0.02	3.0 ± 0.05
50	4.1 ± 0.08	3.0 ± 0.05
100	6.4 ± 0.08	3.1 ± 0.02

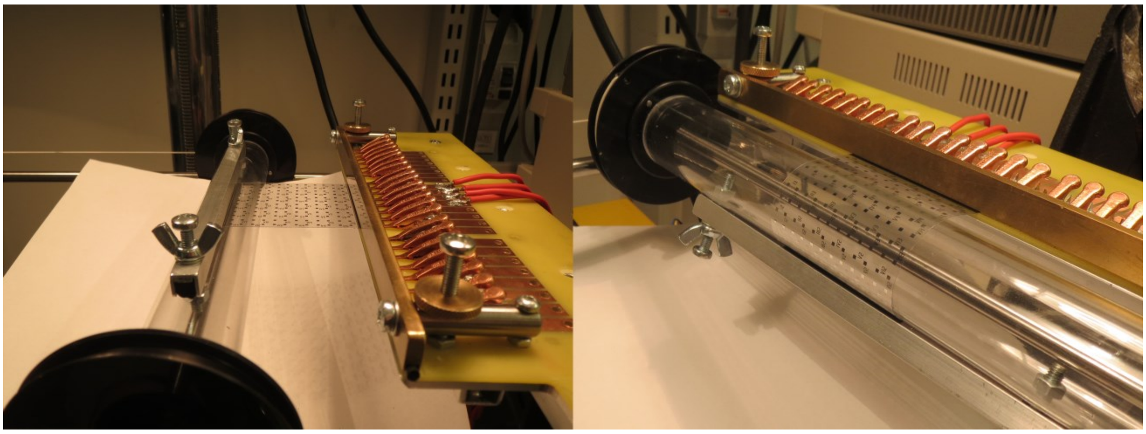

Figure SI-13 Lab-made device for bending test.

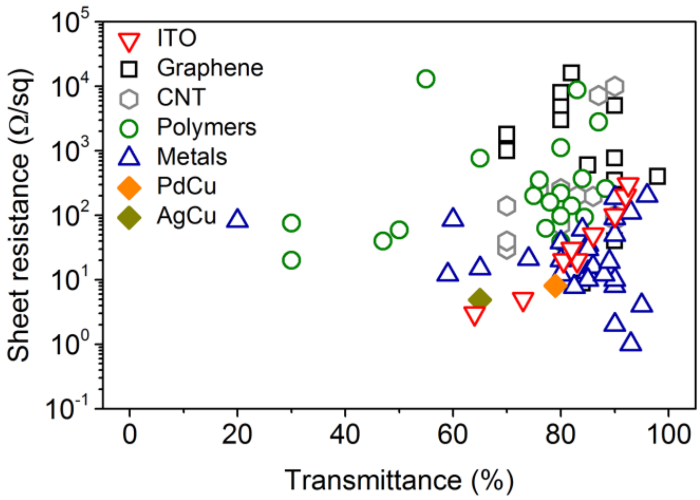

Figure SI-14 Optical transmittance vs. sheet resistance for transparent conductive films based on various structures. Data source: papers listed in Table SI-5-8. Rs and T values of ITO are from J.-Y. Lee, Nano Lett. 2008, 8, 689.

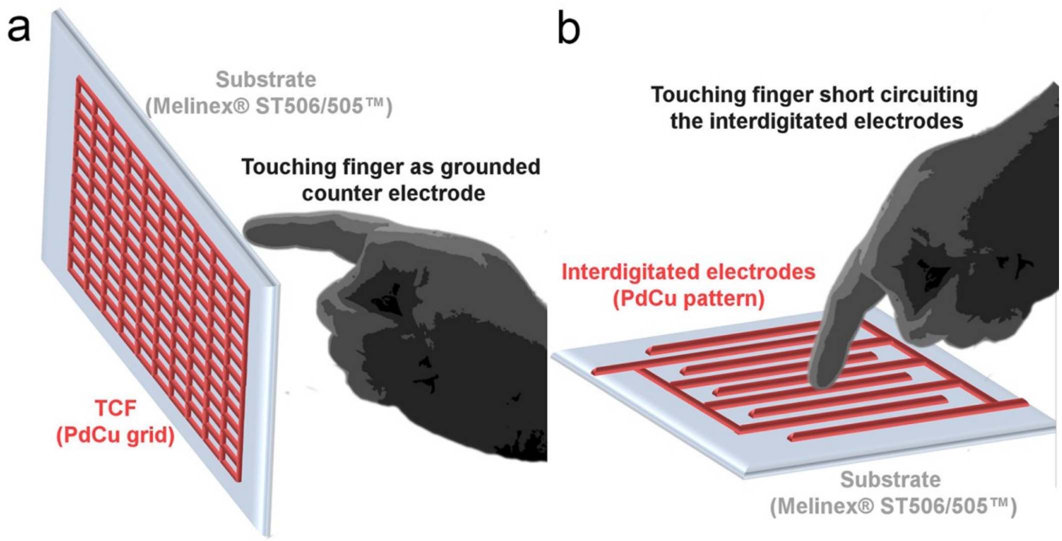

Figure SI-15 Structure schematics of capacitive touch screen (a) and microscopic resistive touch pixel (b).

Table SI-5 Comparison of sheet resistances and optical transmittance of metal or metal nanostructures based transparent conductive films.

	Reference	Material/synthesis	Process	Rs (Ω/sq)	T (%)	λ (nm)
**Ag**	ACS Nano, 2009, 3, 3537.	Ag NP grids	Ink-jet printing, ring-stains	**4 ± 0,5**	**95 ± 3**	400-800
**Ag**	ACS Nano, 2010, 4, 2955.	Ag NWs 50-100 nm width	Mayer rod process	**110-12**	**~93-59**	500
**Ag**	ACS Nano, 2010, 4, 2955.	Ag NWs 30-50 nm width	Mayer rod process	**20**	**~80**	500
**Ag**	ACS Nano, 2009, 3 ,1767.	Ag NWs 85 nm (6.5 µm length)	Vacuum filtration	**13**	**85**	550
**Ag**	Nano Lett., 2008, 8, 689.	Ag NWs PVP, ~100 nm (8.7 µm)	Drop casting	**16**	**86**	400-800
**Ag**	Nano Res., 2010, 3, 564.	Ag NWs	Dry transfer printing	**10**	**85**	550
**Ag**	Sol. Energ. Mat. Sol. C, 2011, 95, 1339	Ag grids	Screen printing	**1**	**~92-94**	
**Ag**	Organic Electronics, 2011, 12, 566	Ag grids	Screen printing	**0.5-15**	**~68-97**	
**AuAg**	Nano Lett., 2009, 9, 4246.	AuAg NWs	Co-red. NWs mesh	**60**	**85**	400-800
**Au**	Langmuir, 2011, 27, 2080.	Au NPs 10 nm	Drop casting on mesh surface	**20**	**82**	400-800
**Cu**	Nano Lett., 2010, 10, 4242	Cu NWs 50-200nm	Electrospinning	**96-80**	**200-12**	300-1100
**Cu**	JACS, 2012, 134, 14283	Cu NWs	vacuum filtration	**90-85**	**90-35**	
**Cu**	Adv. Mater. 2011, 23, 4798	Cu NWs	Meyer rod	**85-90**	**30-186**	550
**Cu**	Adv. Mater. 2010, 22, 3558	Cu NWs	Filtration , printing	**65**	**15**	500
**CuNi**	Nano Lett. 2012, 12, 3193	CuNi NWs		**84**	**60**	550
**Cu**	Nat. Nanotech. 2013, 8, 421	metal nanotrough	Electrospinning and metal deposition	**2**	**90**	300-2000
**Au**	Nat. Nanotech. 2013, 8, 421	metal nanotrough	Electrospinning and metal deposition	**8**	**90**	300-2000
**Ag**	Nat. Nanotech. 2013, 8, 421	metal nanotrough	Electrospinning and metal deposition	**10**	**90**	300-2000
**Ag**	NPG Asia Mater. 2014, 6, e93	nanowires	Filtration onto cellulose nanofiber paper	**12**	**88**	550
**Cu**	NPG Asia Mater. 2014, 6, e105	nanowires	Lactic acid treated, filtration	**19**	**89**	550

Table SI-6 Comparison of sheet resistances and optical transmittance of graphene based transparent conductive films.

Reference	Material/synthesis	Process	Rs (Ω/sq)	T (%)	λ (nm)
Nano Lett., 2008, 8, 323.	Ox/Red	Dip coating	**1800**	**> 70**	500-550
Appl. Phys. Lett., 2008, 92, 263302.	Ox/Red	Spin coating	**5000**	**> 80**	500-550
Nat. Nanotechnol., 2008, 3, 538.	Exfoliating	Langmuir,-Blodgett	**8000**	**80**	500-550
Nano Lett., 2008, 8, 1704.	Exfoliating	Spray deposition	**5000**	**90**	500-550
Nano Lett., 2008, 8, 1704.	Microcleavage	Transfer	**400**	**98**	500-550
Small, 2010, 6, 58.	Exfoliating	Filtration/ transfer	**3000**	**80**	500-550
ACS Nano, 2010, 4, 524.	Ox/Red	Spin coating/ transfer	**1000**	**70**	500-550
Nano Lett., 2009, 9, 30.	CVD Ni	Etching/transfer	**770**	**90**	500-550
Carbon, 2010, 48, 1088.	CVD Ni	Etching/transfer	**600**	**85**	500-550
Nature, 2009, 457, 706.	CVD Ni	Etching/transfer	**280**	**90**	500-550
Nano Lett., 2009, 9, 4359.	CVD Cu	Etching/transfer	**350**	**90**	500-550
arxiv.org/pdf/0912.5485	CVD Cu	Etching/transfer	**40**	**90**	500-550
Adv. Mater. 2012, 24, 2844	Intercalated few-layer graphene		**8.8**	**84**	550
Sci. Reports 2014, 4, 4363	Hydrothermal treatment of MWCNTs	Filtration/transfer	**16000**	**82**	-

Table SI-7 Comparison of sheet resistances and optical transmittance of CNT based transparent conductive films.

Reference	Material/synthesis	Process	Rs (Ω/sq)	T (%)	λ (nm)
Science, 2004, 305, 1273.	Pulsed laser vaporization	Filtration/ transfer	**30**	**> 70**	500-550
Nano Lett., 2006, 6, 1880.	HiPCO	Filtration/ transfer	**7200**	**87**	500-550
	Arc discharge	$SOCl_2$ doping	**380**		500-550
J. Am. Chem. Soc., 2007, 129, 7758.	Arc discharge	Spry coating/ HNO_3 doping	**40**	**70**	500-550
			**70**	**80**	500-550
Phys. Status Solidi RRL, 2007, 1, 178.	HiPCO	Dip coating	**196**	**86**	500-550
Nano Lett., 2008, 8, 1417.	HiPCO	Filtration/ transfer	**140**	**> 70**	500-550
	Laser-ablation				500-550
	Arc discharge				500-550
	Sorting metallic SWCNTs				500-550
Adv. Funct. Mater., 2008, 18, 2548.	Arc discharge	Filtration/ transfer	**170**	**80**	500-550
		Doping ($SOCl_2$ or HNO_3)	**140**		500-550
Chem. Soc. Rev., 2010, 39, 2477.	CVD	Spray coating	**259**	**80**	500-550
		with densification	**150**		500-550
ACS Nano, 2011, 5, 3214.	Ferrocene vapor in CO gas	Filtration and dry transfer	**84**	**90**	550
Phys. Status. Solidi B 2007, 244.4336	SWCNTs/PEDOT:PSS	Inkjet printing	**10000**	**90**	400
ACS Nano 2012, 6 9737	SWNTs/DWNTs	Dip-coating	**100**	**90**	550
Mater. Res. Soc. Symp. Proc., 1537, mrss13-1537-b06-51	SWCNTs		**250**	**76**	550
Jpn. J. Appl. Phys., 2014, 53, 05FD04	SWCNTs	Spray-coated	**200**	**83**	550

Table SI-8 Comparison of sheet resistances and optical transmittance of polymer based transparent conductive films.

Reference	Material/synthesis	Process	Rs (Ω/sq)	T (%)	λ (nm)
Org. Electron., 2009, 10, 1401.	PEDOT:PSS	Spin coating (different thickness)	**2800-160**	**87-78**	400-800
Sol. Energ. Mat. Sol. C, 2006, 90, 123.	PEDOT	Silk screen printing	**20-59**	**30-50**	500
Adv. Funct. Mater., 2004, 14, 615.	PEDOT	Spin coating and polymerization	**140**	**82**	400-700
Org. Electron., 2008, 9, 968.	PEDOT	Spin coating and polymerization	**761-76**	**65-30**	510
Appl. Phys. Lett., 2008, 92, 233308.	PH500 (PEDOT:PSS)	Spin coating	**200**	**75**	400-800
J.Mater. Chem., 2009, 19, 9045.	PEDOT:PSS	Spin-coating	**63-258**	**77.2-88.3**	400-800
Synthetic Met.,2011, 161, 1878	PEDOT:PSS	Spin coating	**13000**	**55**	400-700
Synthetic Met.,2011, 161, 1878	PEDOT:PSS DMSO doping	Spin coating	**40**	**47**	400-700
Synthetic Met.,2011, 161, 1878	PEDOT:PSS/PVP		**350-8830**	**76-83**	400-700
Adv. Mater., 2012, 24, 2436	PEDOT:PSS	Spin coating	**39**	**80**	550
Adv. Funct. Mater. 2013, 23, 3763	PEDOT:PSS	Spin coating	**93**	**84.4**	550
Energy Environ. Sci., 2013, 6, 1956	PEDOT:PSS/glycerol monostearate	Spin coating	**98**	**80**	550

Table SI-9 Parameters for both Pd reactive inkjet printing.

Parameters	Final	Other parameters tested
**Pattern sequence** 1 2 3 4	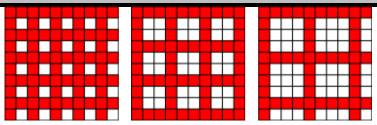	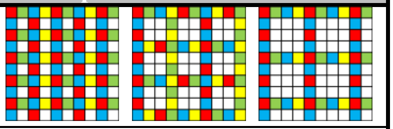
**Cartridge height (mm)**	0.25	0.5; 0.75; 1.00
**Cartridge temperature (°C)**	30	-
**Substrate**	Melinex (ST506/505)	-
**Substrate thickness**	125 µm	-
**Substrate temperature (°C)**	40	25; 30; 45; 60
**Firing voltage (V)**	10-16 V	-
**Meniscus**	0	-
**Jet numbers**	1	-
**Drop spacing (µm)**	20, 25, 30, 100	-
**Layers**	1	1-10

Table SI-10 Parameters for Ag reactive inkjet printing.

Parameters	Final	Other parameters tested
**Pattern sequence** 1 2 3 4	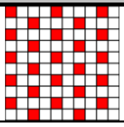	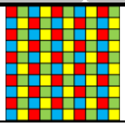
**Cartridge height (mm)**	0.25	0.50
**Cartridge temperature (°C)**	30	40
**Substrate**	Melinex (ST506/505)	-
**Substrate thickness**	125	-
**Substrate temperature (°C)**	60	30; 40; 45; 50
**Firing voltage (V)**	8-14	8-30
**Meniscus**	2	0;1
**Jet numbers**	1	-
**Drop spacing (µm)**	30	25; 100
**Layers**	1	-

Video SI-1 Video of bending test.

Video SI-1.avi

Video SI-2 Video of capacitive screen test.

Video SI-2.avi

Video SI-3 Video of resistive touch pixel test.

Video SI-3.avi
